# The yeast *Geotrichum candidum* encodes functional lytic polysaccharide monooxygenases

**DOI:** 10.1186/s13068-017-0903-0

**Published:** 2017-09-12

**Authors:** Simon Ladevèze, Mireille Haon, Ana Villares, Bernard Cathala, Sacha Grisel, Isabelle Herpoël-Gimbert, Bernard Henrissat, Jean-Guy Berrin

**Affiliations:** 1 INRA, Aix Marseille University BBF, Biodiversité et Biotechnologie Fongiques, 13288 Marseille, France; 2 INRA, UR1268 Biopolymères Interactions Assemblages, 44316 Nantes, France; 3 Architecture et Fonction des Macromolécules Biologiques, UMR7857, CNRS, Aix-Marseille University, 13288 Marseille, France; 4 USC1408, Architecture et Fonction des Macromolécules Biologiques, INRA, 13288 Marseille, France; 5 Department of Biological Sciences, King Abdulaziz University, Jedda, 21589 Saudi Arabia

## Abstract

**Background:**

Lytic polysaccharide monooxygenases (LPMOs) are a class of powerful oxidative enzymes that have revolutionized our understanding of lignocellulose degradation. Fungal LPMOs of the AA9 family target cellulose and hemicelluloses. AA9 LPMO-coding genes have been identified across a wide range of fungal saprotrophs (Ascomycotina, Basidiomycotina, etc.), but so far they have not been found in more basal lineages. Recent genome analysis of the yeast *Geotrichum candidum* (Saccharomycotina) revealed the presence of several LPMO genes, which belong to the AA9 family.

**Results:**

In this study, three AA9 LPMOs from *G. candidum* were successfully produced and biochemically characterized. The use of native signal peptides was well suited to ensure correct processing and high recombinant production of *Gc*LPMO9A, *Gc*LPMO9B, and *Gc*LPMO9C in *Pichia pastoris*. We show that *Gc*LPMO9A and *Gc*LPMO9B were both active on cellulose and xyloglucan, releasing a mixture of soluble C1- and C4-oxidized oligosaccharides from cellulose. All three enzymes disrupted cellulose fibers and significantly improved the saccharification of pretreated lignocellulosic biomass upon addition to a commercial cellulase cocktail.

**Conclusions:**

The unique enzymatic arsenal of *G. candidum* compared to other yeasts could be beneficial for plant cell wall decomposition in a saprophytic or pathogenic context. From a biotechnological point of view, *G. candidum* LPMOs are promising candidates to further enhance enzyme cocktails used in biorefineries such as consolidated bioprocessing.

## Background

Lignocellulosic biomass is a central resource for biofuel and bio-based industries. Increasing the recovery yields of the monosaccharide during lignocellulose conversion is one of the main bottlenecks of the biofuel production process due to the complex structure of lignocellulosic material limiting enzymatic digestibility [1, 2]. Lytic polysaccharide monooxygenases (LPMOs), a new class of microbial enzymes, were identified as boosters of biomass deconstruction through the oxidative cleavage of polysaccharides increasing the activity of cellulases [3]. Their significant contribution to the saccharification of biomass led to their incorporation in last-generation commercial enzyme cocktails [4]. LPMOs are secreted enzymes [5], and signal peptide removal uncovers the N-terminal histidine. This histidine forms part of a histidine brace that holds the copper ion, which is critical for enzymatic activity.

In the CAZy database, LPMOs are placed within the Auxiliary Activity (AA) enzyme class [6] in families AA9, AA10, AA11, and AA13 [7, 8]. In filamentous fungi, AA11 LPMOs cleave chitin [9] and AA13 target starch [10], while AA9 members target cellulose and hemicelluloses [11]. In May 2017, out of 3400 members belonging to the AA9 LPMO family [12], the 26 characterized ones all originated from filamentous fungi. The substrate specificities of some of these AA9 LPMOs are restricted to cellulose [13], while others display a broader substrate specificity toward xyloglucans and/or beta-glucans in addition to cellulose [14–17]. A few of them are also active on soluble cello-oligosaccharides [15, 18, 19]. The LPMO9H from *Podospora anserina* has been shown to create nicking points on cellulose microfibrils triggering the disintegration of the cellulose fibrillar structure through modification of accessible and inaccessible surfaces of the fibrils [20].

AA9 LPMO-coding genes have been identified across Ascomycota and Basidiomycota including wood-rotting fungi (white rots and brown rots); litter degraders; symbiotic, endophytic fungi; and fungal pathogens [21]. However, so far they have not been found in more basal lineages. The yeast *Geotrichum candidum* (teleomorph = *Galactomyces candidus*) belongs to the Saccharomycotina subphylum within the Ascomycota phylum in the Kingdom of Fungi. It compromises most of the ascomycete yeasts (e.g., *Saccharomyces cerevisiae*). *G. candidum* is well known for its creamy white color it creates on the rind of brie and camembert, and is able to grow on wooden cheese boxes [22]. Some strains of *Geotrichum candidum* display high cellulolytic and xylanolytic activities and can degrade efficiently filter paper and cotton. A GH7 cellobiohydrolase, which was found to be the predominant protein in *G. candidum* secretome, was characterized and shown to have interesting biochemical properties compared with canonical *Trichoderma reesei* cellobiohydrolases, such as a broader pH range and reduced product inhibition [23]. Recently, genomic analysis of the strain *G. candidum* CLIB 918 revealed the presence of four genes encoding AA9 LPMOs [24]. Together with four GH45 endoglucanases, these AA9 LPMO genes have been specifically retained by *G. candidum* after the filamentous fungi–yeasts split concomitant with the yeasts’ genome contraction, thus contributing to the phenotypic specificity of lineages [24]. The unique arsenal of carbohydrate-degrading enzymes of *G. candidum* compared with other yeasts could be involved in plant cell wall degradation in a saprophytic or pathogenic context but experimental investigations are necessary to validate the hypothesis. In this study, we investigated the substrate specificities and contribution to biomass degradations of three *G. candidum* AA9 LPMOs.

## Results

### Production of *G. candidum* AA9 LPMOs

The genes products encoding *Gc*LPMO9A (GECA32s02892g), *Gc*LPMO9B (GECA32s00307g), *Gc*LPMO9C (GECA21s01121g), and *Gc*LPMO9D (GECA08s04487g) are all formed by a single AA9 catalytic module starting with the canonical-conserved histidine residue common to all LPMOs. The AA9 modules are preceded by around 18–20 residues’ signal peptides and carry a C-terminal extension (Table 1). The overall amino acid sequence similarity of the *G. candidum* AA9 LPMOs with each other is relatively high, ranging from 47 to 67%, in spite of wide variations in sequence length, which ranges from 288 residues (*Gc*LPMO9A) to 530 residues (*Gc*LPMO9C). To produce the recombinant proteins in *Pichia pastoris*, the C-terminal sequences were removed. An important feature to consider upon production of recombinant LPMOs is the perfect processing of the signal peptides during secretion to ensure correct binding of the catalytic copper ion by the N-terminal histidine residue. Optimal processing of signal peptides during heterologous production is protein dependent, and heterogeneity on N-terminal sequences is a recurrent problem [25]. In *P. pastoris*, the use of the α-mating factor (α-MF) as signal peptide is sometimes associated with incorrect cleavage by the Ste13 protease [26]. Therefore, we designed plasmid constructs using both the α-MF and the native signal sequences of each *G. candidum* LPMOs to foster recombinant protein production in *P. pastoris*. Using this strategy, *Gc*LPMO9A, *Gc*LPMO9B, and *Gc*LPMO9C were successfully produced and purified to homogeneity. However, *Gc*LPMO9D was not detected in the supernatant of *P. pastoris* after 3 days of induction and was thus not further investigated. SDS-PAGE analysis revealed that *Gc*LPMO9A, *Gc*LPMO9B, and *Gc*LPMO9C each displayed an apparent molecular weight higher than the expected ones. Indeed, 6-12 *N*-glycosylation sites (Table 1) are predicted on the sequences, and consistently, endoH treatment allowed for the release of proteins, with the decreasing apparent molecular mass, closer to the expected values, indicating that the proteins are *N*-glycosylated. In addition, mass spectrometry analysis revealed a heterogeneous mass shift of about 3–5 kDa compared to the predicted mass for *Gc*LPMO9A, indicating a heterogeneous glycosylation pattern (theoretical mass: 26.9 kDa, observed values: 29–32 kDa). Replacing the α-MF by the native signal peptide of the proteins resulted in contrasting production yields. Using the α-MF, production yields were over 100 mg l^−1^ of purified protein of culture for all protein*s*, except for *Gc*LPMO9A, which reached around 60 mg l^−1^. Production yields significantly increased when native signal sequences of *Gc*LPMO9A and *Gc*LPMO9B were used (Table 1). Besides the fact that the proteins are expressed to high levels, these results highlight that the choice of the signal peptide strongly affects the production yield of recombinant proteins, a general feature that remains strongly empirical. In addition, N-terminal sequencing indicated that the nature of the signal peptide used massively affected their processing by *P. pastoris*. Indeed, using the α-MF, 84% of *Gc*LPMO9A, 73.5% of *Gc*LPMO9B, and 79% of *Gc*LPMO9C kept the EAEA sequence upstream the His1 residue, indicating a partial signal peptide processing. Indeed, signal peptide cleavage is achieved by the subsequent actions of the two proteases Kex2 and Ste13. Ste13 cleavage site is right downstream this EAEA tetrapeptide, which is often retained in the case of high levels of secreted recombinant proteins expression in *P. pastoris*. The expected sequence starting with Histidine 1 accounted only for 5% for all three proteins. However, using their native signal peptides, the presence of the desired sequence was found at 10, 90, and 63% for *Gc*LPMO9A, *Gc*LPMO9B, and *Gc*LPMO9C, respectively.

**Table 1:** Production and activity details of AA9 LPMO used in this study

Protein	Modularity	Predicted *N*-glycosylation sites	Signal peptide	Production yield (g/L)	Specific activity (U/g)^a^	Total activity (U/L)	Competitive assay (% residual activity)	
							PASC (0.1%)	Xyloglucan (0.1%)
*Gc*LPMO9A	1-20[SP]-21-243[AA9]-244-288[Cterm]	6	α-factor	0.062	0.174	0.011	ND	ND
			Native	0.110	0.286	0.031	33.9 ± 5.4	16.3 ± 0.4
*Gc*LPMO9B	1-18[SP]-19-244[AA9]-245-359[Cterm]	10	α-factor	0.113	0.011	0.001	ND	ND
			Native	0.194	1.012	0.196	24.0 ± 0.6	15.1 ± 3.8
*Gc*LPMO9C	1-18[SP]-19-242[AA9]-243-530[Cterm]	12	α-factor	0.150	0.020	0.003	ND	ND
			Native	0.072	0.474	0.034	ND	ND

*SP* signal peptide, *ND* not determined, *nd* not detected, *AA9* AA9 catalytic module

^a^Specific activity was determined using amplex red assay. *N*-glycosylation sites were predicted using the Hirst group prediction server [40]

### GcLPMO9A and GcLPMO9B are active toward cellulose and xyloglucan

In order to verify the copper loading of the histidine brace of *Gc*LPMO9s, the enzymes were assayed for assessing their ability to produce H_2_O_2_ using ascorbate as electron donor [15, 27]. In the absence of substrate, enzymes were able to produce H_2_O_2_ with various efficiencies (Table 1). The use of the native signal peptide increased the specific activities by 92- and 24-folds for *Gc*LPMO9B and *Gc*LPMO9C, respectively, compared to those the same proteins with the α-MF. *Gc*LPMO9B was the most affected by signal peptide replacement, as both the specific activity and the production yield were increased, resulting in an overall 159-fold increase, from 1.24 U/l with the α-MF up to 197 U/l with the native signal peptide. This increase can be correlated with a better cleavage of the signal peptides. Indeed, upon production with their native signal peptides, the vast majority of the protein samples harbored the correct His1 N-term residue (up to >90% for *Gc*LPMO9C), while those with the α-MF were badly processed (below 5%). This percentage of correctly processed proteins was rationally correlated with the activity, as *Gc*LPMO9B was found to be the most active, followed by *Gc*LPMO9C (63% of His1), and finally by *Gc*LPMO9A (10%). Therefore, N-terminal sequencing and H_2_O_2_ production should be systematically performed prior to functional characterization of LPMOs.

**Fig. 1:**
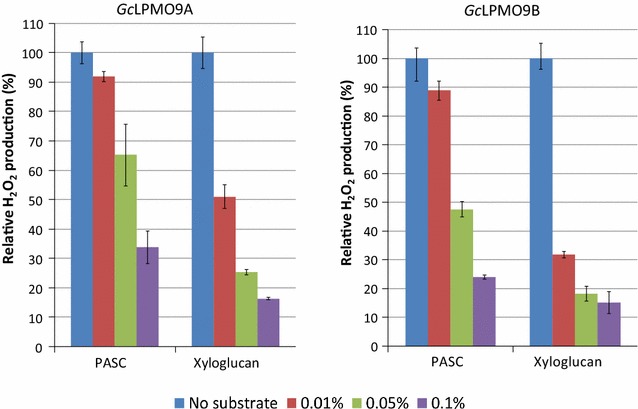
Relative H_2_O_2_ productions of *Gc*LPMO9A and *Gc*LPMO9B in the absence and the presence of increasing amounts of PASC and xyloglucan. Relative H_2_O_2_ productions of *Gc*LPMO9A (left) and *Gc*LPMO9B (right) in the absence or the presence of 0.01, 0.05, and 0.1% of PASC or xyloglucan. Error bars indicate standard deviations from triplicate independent experiments

In order to identify the putative natural substrates of these enzymes, H_2_O_2_ production was measured in the presence of a range of plant polysaccharides. The redox copper center of LPMOs can catalyze the formation of hydrogen peroxide in the absence of any substrate when a suitable electron donor is provided. Therefore, a decrease in H_2_O_2_ production is usually observed in the presence of the substrate. Among all the polysaccharides tested, decreases in H_2_O_2_ production were observed for *Gc*LPMO9A and *Gc*LPMO9B on phosphoric acid-swollen cellulose (PASC) by 33.9 ± 5.4% and 24.0 ± 0.6%, respectively; and on xyloglucan with residual activities by 16.3 ± 0.4% and 15.1 ± 3.8%, respectively, demonstrating an enzyme dose-dependent behavior (Fig. 1). The activity profile was not significantly affected by the nature of the signal peptide—PASC and xyloglucan remaining the only two hits (Table 1). No decrease in H_2_O_2_ production was observed with *Gc*LPMO9C.

**Fig. 2:**
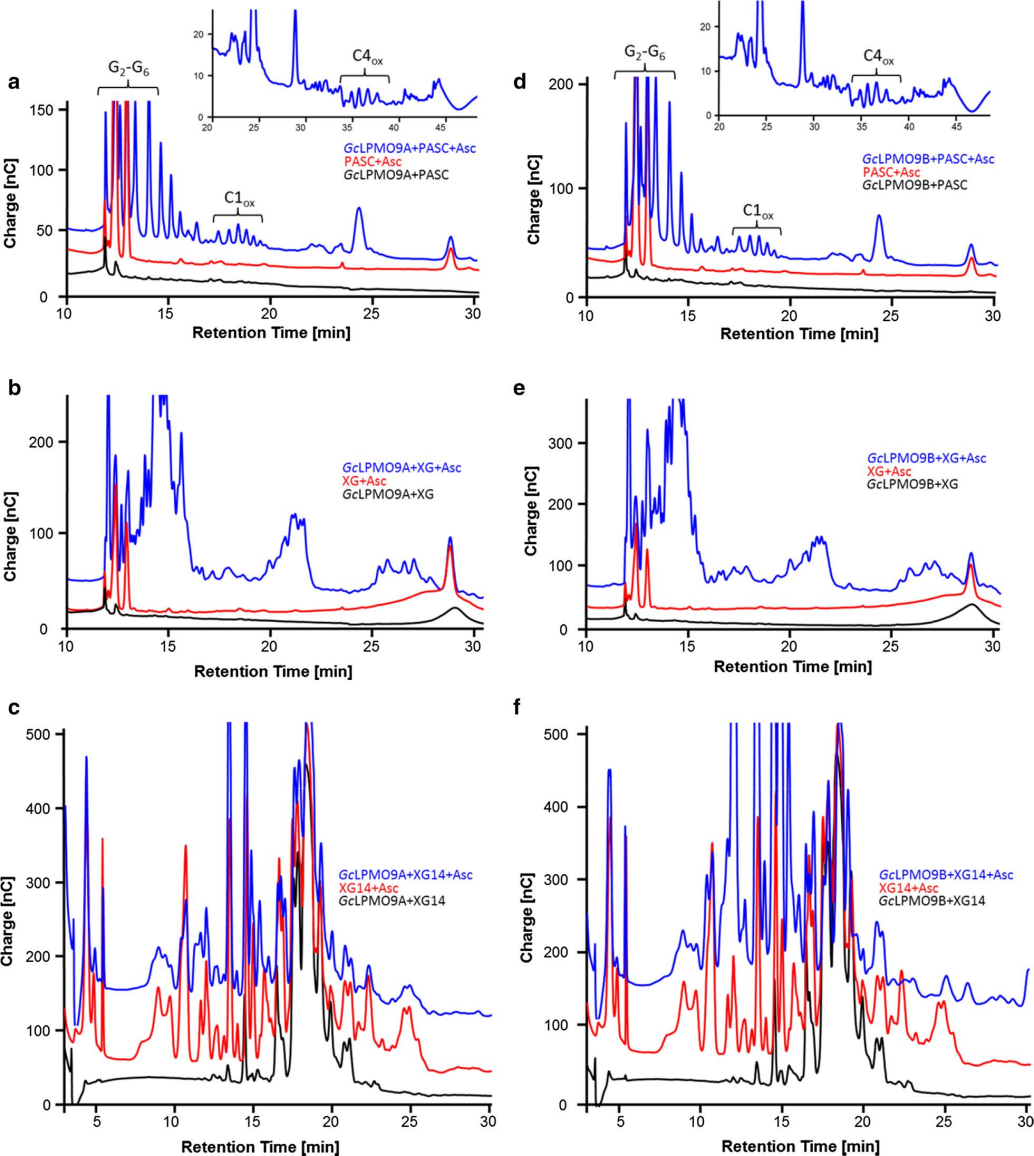
Analysis of products released from PASC, xyloglucan, and xyloglucan oligosaccharides XG14 by *Gc*LPMO9A and *Gc*LPMO9B. HPAEC chromatograms show the products released by *Gc*LPMO9A from PASC (**a**), xyloglucan (**b**), XG14 (**c**), and by *Gc*LPMO9B from PASC (**d**), xyloglucan (**e**), and XG14 (**f**). Enzyme assays in the presence of substrate and ascorbate are shown in blue and control reactions without ascorbate and without enzyme are shown in black and red, respectively. The insets in **a** and **d** highlight the releases of C4 and C1-C4-oxidized cello-oligosaccharides by *Gc*LPMO9A and *Gc*LPMO9B, respectively. PASC, phosphoric acid-swollen cellulose; XG, Xyloglucan

*Gc*LPMO9A and GcLPMO9B were further assayed using ionic chromatography in order to confirm oxidative cleavage of cellulose and xyloglucan. On cellulose, chromatograms showed that both enzymes were able to release a series of nonoxidized cello-oligosaccharides of DP2–DP6, (retention times between 10 and 17 min) together with a series of peaks (retention times between 17 and 20 min) attributable to C1-oxidized cello-oligosaccharides, according to C1-oxidized standards (Fig. 2). In addition, other oxidized species were observed at 25 min and between 35 and 38 min, as well as peaks around 40–44 min, which correspond to C4-oxidized and C1–C4-double-oxidized products, respectively, based on previous analyses with respect to *Podospora anserina* AA9 LPMOs [15]. All these peaks were not detected without ascorbate, demonstrating that both *Gc*LPMO9A and *Gc*LPMO9B require an exogenous electron donor. Care was taken to discriminate the peaks that were due to the action of ascorbate, well known for inducing basal release of oligosaccharides, from those directly attributable to the action of the enzymes.

*Gc*LPMO9A and *Gc*LPMO9B also had comparable degradation profiles when assayed on xyloglucan (Fig. 2). Both enzymes released a broad range of xyloglucan oligosaccharides eluting from 12 to 35 min, some of them matching those released by *Pa*LPMO9H that releases C1-, C4-, and C1–C4-oxidized xyloglucan oligosaccharides [17]. When assayed on XG14, *Gc*LPMO9A and *Gc*LPMO9B released appreciable amounts of mixed branched oligosaccharides, some substrate peaks being reduced while products appeared, eluting from 10 to 35 min. Some of the peaks could be attributed to XG7, and *Gc*LPMO9B clearly appeared to be the more active of the two LPMOs (Fig. 2). In-depth characterizations of the enzymes’ activities on such a complex substrate remain to be conducted using mass spectrometry [17], as many of the detected peaks (apart from those pointed here) are also present under the ascorbate condition.

### GcLPMO9s improve the deconstruction of plant biomass

**Fig. 3:**
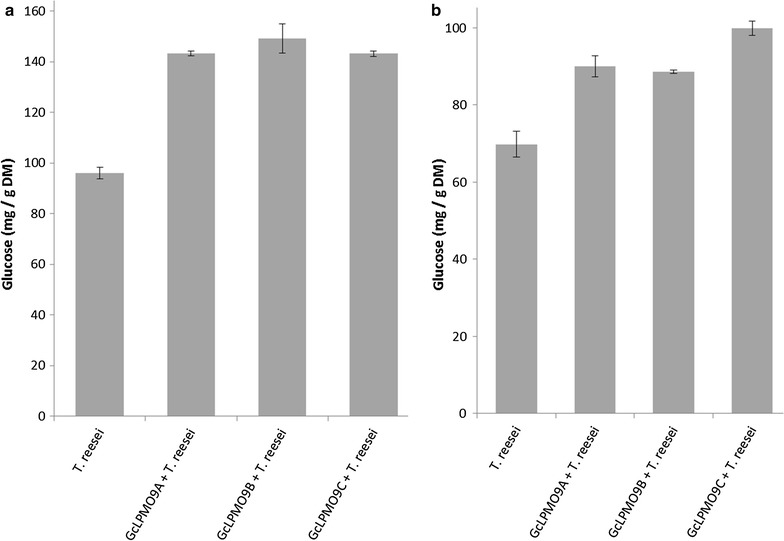
Contribution of *Gc*LPMO9 enzymes to the saccharification of woody biomass. Glucose releases upon saccharification of birchwood (**a**) and poplar (**b**) by the *Trichoderma reesei* enzyme cocktail in the presence of 2.2 µM *Gc*LPMO9 enzymes. All assays were run in the presence of 1 mM ascorbic acid. Glucose was quantified using HPAEC. Error bars indicate standard deviations from triplicate independent experiments

In order to assess the contribution of *Gc*LPMO9s on woody biomass degradation, these enzymes were tested using a commercial *T. reseei* cellulases cocktail on the saccharification of birchwood fibers and pretreated poplar. Assays were performed sequentially because we believe that LPMOs act first as observed in a time-course study of the fungus *Laetisaria arvalis* where LPMOs are secreted before hydrolases [28]. Quantification of the total glucose released at the end of the reactions clearly showed that each LPMO had a comparable boosting effect on birchwood fibers and poplar (Fig. 3). Compared with the *T. reesei* enzyme cocktail alone, glucose releases were improved by 49, 55, and 49% on birchwood fibers, and by 29, 27, and 43% on poplar, by *Gc*LPMO9A, *Gc*LPMO9B, and *Gc*LPMO9C, respectively.

**Fig. 4:**
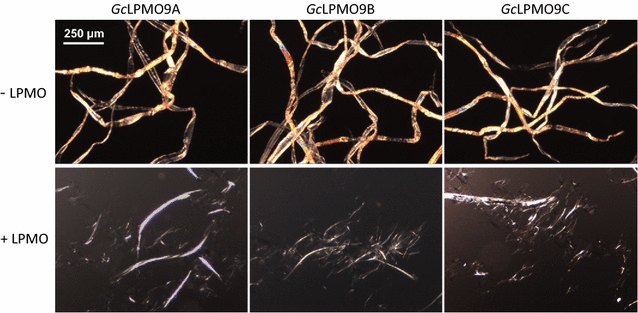
Microscopic observation of cellulose fibrils disruption. Pictures showing the activities of *Gc*LPMO9A, *Gc*LPMO9B, and *Gc*LPMO9C on cellulose fibrils. The fibrils before (top) and after treatment (bottom) with 20 mg g^−1^ LPMO in the presence of 1 mM ascorbic acid for 48 h at 40 °C. Images were recorded after dispersion and the representative of the samples was analyzed

To get further insights into the mode of action of *Gc*LPMO9s, we directly assessed their disruptive action on birchwood fibers using microscopy. Each *Gc*LPMO9 enzyme in the presence of ascorbate led to the disruption of the cellulose fibrils in a similar way to the one that was previously observed with *Pa*LPMO9H [20], suggesting that *Gc*LPMO9s create nicking points in which rupture of chains release elementary nanofibrils (Fig. 4). Taken together, these results clearly demonstrate that the LPMOs encoded by *G. candidum* genome are functional and contribute to plant biomass degradation.

## Discussion

**Fig. 5:**
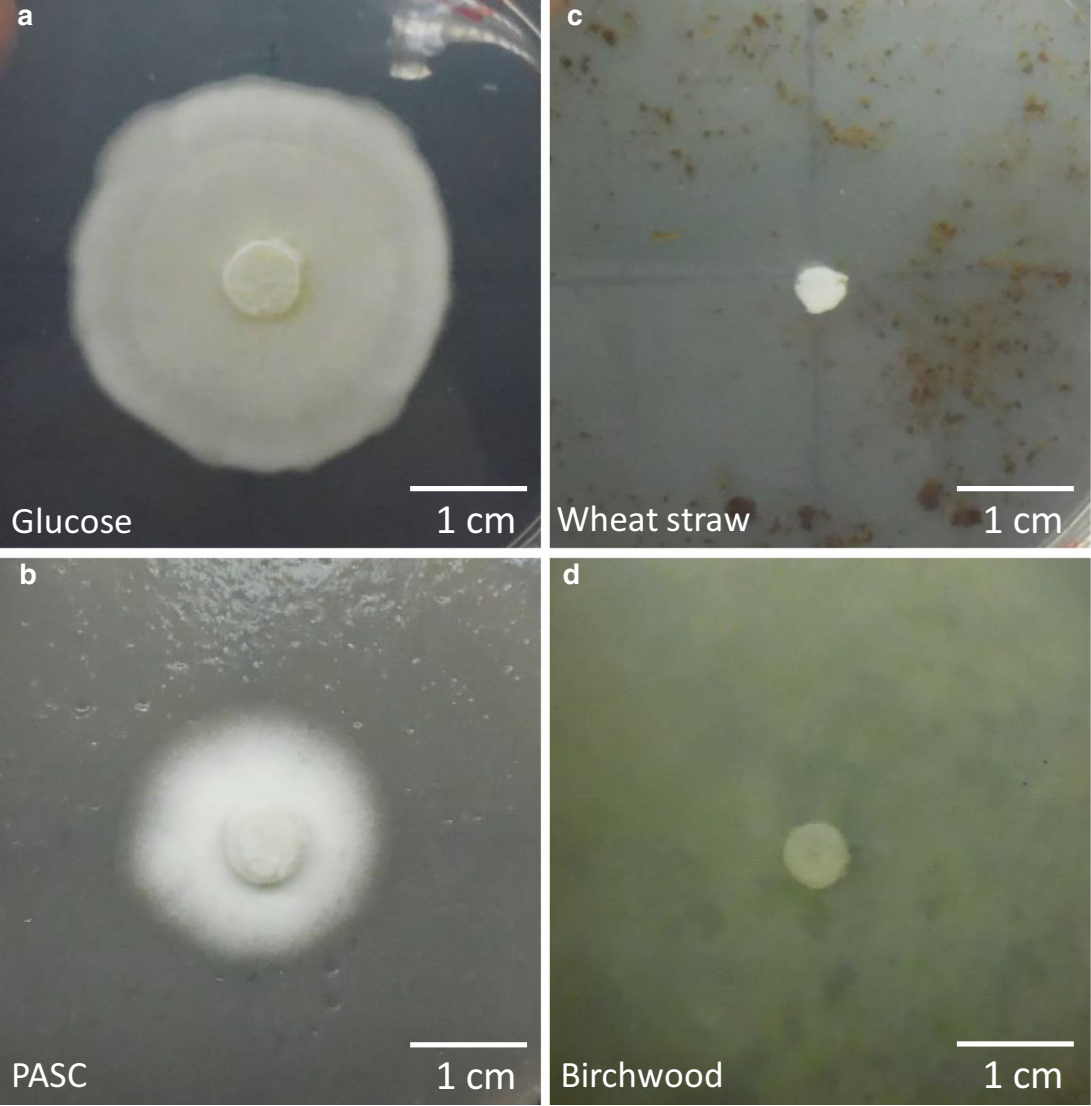
Growth of *G. candidum* CLIB 918 on diverse carbon sources. Pictures show the propagation of *G. candidum* CLIB 918 from the central inoculate disk onto glucose (**a**), PASC (**b**), wheat straw (**c**), and birchwood (**d**). The plates shown here are those from the condition in the absence of ascorbate. Identical profiles were observed in the presence of 1 mM ascorbate. The pictures were taken after 7 days of incubation at 30 °C

The presence of AA9 LPMO genes in *G. candidum* was unexpected from an organism selected for cheese preparation. Apart from cheese, this yeast can be found elsewhere in nature, and particularly, some strains have been described as plant and fruit pathogens or degraders [29]. On the other hand, several strains of *G. candidum*, such as *G. candidum* 3C, are well known for their cellulose-degrading capabilities [23]. In order to confirm the cellulolytic behavior of the strain CLIB 918, we assessed its growth capabilities on different carbon sources (Fig. 5). Under our experimental conditions, *G. candidum* CLIB 918 was able to grow on cellulose, but the growth was not correlated to the presence of ascorbic acid, a typical electron donor of LPMOs (data not shown). The lack of growth on wheat straw and birchwood could be explained by the absence of lignin-degrading enzymes in *G. candidum* genome.

Our study reveals that the AA9 LPMOs of yeast origin characterized for the first time are functional enzymes acting on plant polysaccharides. Striking differences between *Gc*LPMO9 enzymes were observed. Indeed, only *Gc*LPMO9A and *Gc*LPMO9B released soluble C-1 and C-4-oxidized oligosaccharides, while all three *Gc*LPMO9 enzymes disrupted cellulosic fibers and boosted biomass conversion, suggesting a difference in terms of substrate specificity. We believe that amorphous cellulose is not a suitable substrate for *Gc*LPMO9C, which may act at the surface of cellulose microfibrils without any release of soluble products. Most biochemical methods used to assess the activity of LPMOs are based on the release of short oligosaccharides and might not be adaptable to all LPMOs. Therefore, the impact of LPMOs on the insoluble part of the fibers should be systematically implemented into the assessment of their activity.

From a biotechnological point of view, *Gc*LPMO9 enzymes are promising enzymes for applications in a biorefinery context. Replacing the typical α-MF signal peptide by the native signal peptide was beneficial, increasing the production yields of active enzymes in *P. pastoris*. The use of this host opens the way to achieving the production of large amounts of active recombinant LPMOs, a critical point for industrial applications. Yet, as no rule-of-thumb exists regarding the prediction of the best signal peptide for a specific target protein, larger-scale productions still have to be conducted in bioreactors to optimize production yields. *G. candidum* AA9 LPMOs could be appropriate enzymes for consolidated bioprocessing (CBP), which combines enzyme production, saccharification, and fermentation in a single step [30]. One of the strategies for engineering a CBP biocatalyst is to develop recombinant yeast strains displaying cellulases and hemicellulases on the cell surface [31, 32]. Yeast genomes are generally poor in cellulose-acting enzymes, and only a few CAZymes of yeast origin have been so far characterized [33, 34].

## Conclusion

The AA9 LPMOs characterized for the first time in this study are those of yeast origin. The use of their native signal peptides facilitated their functional production in *P. pastoris*. All three enzymes disrupted cellulose fibers and improved the saccharification of woody biomass. *Gc*LPMO9A and *Gc*LPMO9B were both active on cellulose and xyloglucan, releasing a mixture of soluble C1- and C4-oxidized oligosaccharides from cellulose. The unique enzymatic arsenal of *G. candidum* compared with other yeasts could be more beneficial for plant cell wall decomposition in a saprophytic or pathogenic context. In biorefineries, *G. candidum* LPMOs are promising candidates to further enhance enzyme cocktails. These AA9 LPMOs of yeast origin are perfectly suited for CBP, in which the engineered yeast strains should produce efficiently these LPMOs, which are able to improve the conversion of plant biomass when used in concert with cellulases and hemicellulases.

## Methods

### Cloning and production of Geotrichum candidum LPMOs

Protein sequences of *Gc*LPMO9A (GenBank ID CDO58049), *Gc*LPMO9B (GenBank ID CDO57961), and *Gc*LPMO9C (GenBank ID CDO57431) were used for generating codon-optimized DNA sequences for production in *P. pastoris*. Total gene synthesis (Genescript, Piscataway, USA) was performed prior to cloning into the pPICZαA expression vector (Invitrogen, Cergy-Pontoise, France). Care was taken in order to clone the ORF in frame with both the yeast α-MF and the C-term-(His)_6_ tag encoding sequences while removing the native-predicted signal peptide and the C-term-predicted disordered region from the final secreted protein sequences (Table 1). Alternative constructs where the yeast α-MF from the expression vector was swapped with the native signal peptide were also designed. *Pme*I-linearized plasmids were used for transformation into electrocompetent *P. pastoris* X33 cells as described in Couturier et al. [35]. Zeocin-resistant *P. pastoris* transformants were then screened for optimal protein production. The best-producing transformants were grown in 1 L of BMGY containing 1 ml l^−1^ of PTM_4_ salts (2 g l^−1^ CuSO_4_·5H_2_O; 3 g l^−1^ MnSO_4_·H_2_O; 0.2 g l^−1^ Na_2_MoO_4_·2H_2_O; 0.02 g l^−1^ H_3_BO_3_; 0.5 g l^−1^ CaSO_4_·2H_2_O; 0.5 g l^−1^ CoCl_2_; 12.5 g l^−1^ ZnSO_4_·7H_2_O; 22 g l^−1^ FeSO_4_·7H_2_O; biotin 0.2 g l^−1^; and concentrated H_2_SO_4_ 1 ml) in shaken flasks at 30 °C in an orbital shaker (200 rpm) for 16 h to an OD_600_ of 2–6. Expression was induced by transferring the cells into 200 ml of BMMY containing 1 ml l^−1^ of PTM_4_ salts at 20 °C in an orbital shaker (200 rpm) for another 3 days. Each day the medium was supplemented with 3% (v/v) methanol.

### Purification of Geotrichum candidum LPMOs

The culture supernatants were recovered by pelleting the cells by centrifugation at 2700 × *g* for 5 min, 4 °C and filtered on 0.45-µm filters (Millipore, Molsheim, France) to remove any remaining cells. After adjusting the pH to 7.8, the supernatants were filtered once more on 0.2-µm filters and loaded onto 5-ml Histrap columns (GE healthcare, Buc, France) connected to an Akta Xpress system (GE healthcare). Prior to loading, the columns were equilibrated in Tris HCl 50 mM pH 7.8; NaCl 150 mM (buffer A). The loaded columns were then washed with 5 column volumes (CV) of 10 mM imidazole in buffer A, before the elution step with 5 CV of 150 mM imidazole in buffer A. After elution, the fractions containing the purified proteins were pooled, and buffer was exchanged with Tris HCl pH 7.8, NaCl 50 mM using PD-10 columns (GE healthcare). An aliquot of each fraction was loaded onto an SDS-PAGE stain-free gel (Bio-rad, Marnes-la-Coquette, France) to check protein purity. Protein concentrations were determined using a Nanodrop ND-2000 spectrophotometer (Thermo Fisher Scientific, IL, USA) and theoretical masses and molar extinction coefficients calculated from protein sequences.

### Amplex red assays

A fluorimetric assay based on Amplex red and horseradish peroxidase was used as described previously [15, 27]. One hundred microliter reactions containing 50 mM sodium acetate pH 6.0, 50 μM Amplex Red (Sigma-Aldrich, Saint-Quentin Fallavier, France), 7.1 U ml^−1^ horseradish peroxidase, 1–10 μM purified *Gc*LPMOs, and 50 μM ascorbate as reductant in water were incubated for 30 min at 30 °C in 96-Well Black Solid Plates (Greiner Bio One, Kremsmünster, Austria). Fluorescence was measured using excitation and emission wavelengths of 560 and 595 nm, respectively, using a Tecan Infinite M200 plate reader (Tecan, Männedorf, Switzerland). The specific activity was calculated from an H_2_O_2_ calibration curve, and the slope (13,227 counts μmol^−1^) was used to convert the fluorimeters readout (counts min^−1^) into enzyme activity. Various polysaccharides (PASC, CMC, avicel, xylan, xyloglucan, chitin, lichenan, curdlan, starch, β-1,3-glucan, glucomannan, and cello-pentaose) were tested to a final concentration of 0.1% (w/v) or 0.4 mg ml^−1^. For dose–response inhibition assays on PASC and xyloglucan, substrates were added to final concentrations of 0.1, 0.05, and 0.01% (w/v). All measurements were performed in triplicates.

### Cellulose and xyloglucan degradation assays

All reactions were performed in 1.5-mL tubes. Two hundred microliters reaction volumes containing 0.1% (w/v) PASC or xyloglucan, 1 mM ascorbate as electron donor, 10 µM LPMO in 50 mM sodium acetate pH 5, 50 mM NaCl were incubated for 24 h at 40 °C, 800 rpm in a thermomixer (Eppendorf, Montesson, France). PASC was prepared from Avicel as described by Wood et al. [36]. Tubes were then heated at 100 °C for 10 min to stop the reactions, and centrifuged for 10 min at 13,000 × *g* in order to separate the supernatant from the insoluble material.

### Analysis of degradation products

Mono-, oligosaccharides and their corresponding aldonic acid and C4-gemdiol forms generated after PASC cleavage were analyzed by high-performance anion exchange chromatography coupled with amperometric detection (HPAEC-PAD) as described by Westereng et al. [37] using nonoxidized cello-oligosaccharides (Megazyme, Wicklow, Ireland) as standards. Oligosaccharides standards oxidized at the C1 position were produced from nonoxidized cello-oligosaccharides using a cellobiose dehydrogenase as described in Bennati-Granier C. et al. [15]. For analysis of xyloglucan degradation products, purified xylo-oligosaccharides of known structure XXXG, XXLG, and XLLG according to the nomenclature of Fry et al. [38] were used as standards (Megazyme reference O-XGHON).

### Growth of Geotrichum candidum CLIB 918 on cellulosic substrates

The *G. candidum* strain CLIB 918 used in the present study was provided by the CIRM-levures (INRA, Micalis Institute, Thiverval-Grignon, France). Cellulosic degradation capabilities of *G. candidum* CLIB 918 were assessed on various substrates (PASC, native-milled wheat straw, and birchwood fibers) by inoculating agar plates containing each substrate as sole carbon source. Each plate contained a 1.5% agar bottom layer and a top layer containing 1.5% agar, 1.5% substrate, 0.17% YNB, and 40 mM di-ammonium tartrate. A positive control with glucose as sole carbon source was added. Similar plates containing an additional 1 mM ascorbic acid were also prepared. A pregrown YNB-agar plate of *G. candidum* was used to inoculate a 6 mm disk at the center of each plate. Growth of *G. candidum* from this central disk was monitored over 7 days of incubation at 30 °C.

### Saccharification assays

The enzymatic treatments were carried out in sodium acetate buffer (50 mM, pH 5.2) in a final volume of 1 ml at 0.5% consistency (w d.m./v). The two assayed substrates were birchwood fibers and acid-pretreated poplar. Birchwood fibers mainly consisted of cellulose. The total carbohydrate contents (% d.w.) of poplar were 50.85 ± 0.91 of glucose, 0.39 ± 0.01 of xylose, and 0.07 ± 0.01 of arabinose. The LPMO treatment was carried out sequentially using a CL847 *T. reesei* enzyme cocktail [39]. Each *Gc*LPMO9 enzyme was added to the substrates at a concentration of 2.2 µM in the presence of 1 mM ascorbic acid for 72 h, followed by the addition of 1 mg g^−1^ d. m. substrate of commercial cellulases from *T. reesei* for 24 h. Control experiments were performed in the absence of LPMO in a buffer containing 1 mM ascorbate. Enzymatic treatments were performed in 2-ml tubes incubated at 45 °C and 850 rpm in a rotary shaker (Infors AG, Switzerland). Then, samples were centrifuged at 13,000 × *g* for 5 min at 4 °C, and the soluble fraction was heated for 10 min at 100 °C to stop the enzymatic reaction. Glucose was quantified using high-performance anion exchange chromatography coupled with amperometric detection (HPAEC-PAD) as described in Westereng et al. [37]. In brief, the eluents were 0.1 M NaOH (eluent A) and 1 M NaOAc in 0.1 M NaOH (eluent B). Elution was performed onto a CarboPac PA1 column 2 × 250 mm at a constant flow rate of 0.25 ml min^−1^ at 30 °C, using a linear gradient of 0–10% eluent B over 10 min, 10–30% eluent B over 25 min, and an exponential gradient of 30–100% eluent B over 5 min. The initial condition (100% eluent A) was then restored in 1 min and maintained for 9 min.

### Mass spectrometry

Matrix-assisted laser desorption ionization mass spectra analyses were performed on a Microflex II mass spectrometer (Bruker Daltonics, MA, USA). One μL of matrix [10 mg of 2,5-Dihydroxybenzoic acid in 1 ml of CH_3_CN/H_2_O 50/50 (v/v), 0.1% formic acid (v/v)] was added to 1 μL of intact LPMO protein sample (100 pmol) in the same solution. Then, the mixture was allowed to dry at room temperature. Data acquisition was operated using the Flex control software. External mass calibration was carried out on Peptide calibration standard (Bruker Daltonics).

### Microscopic observation of cellulose fibrils

Aqueous dispersions of Kraft birchwood cellulosic fibers (kindly provided by Sandra Tapin, FCBA, Grenoble, France) were adjusted to pH 5.2 with acetate buffer (50 mM) in a final reaction volume of 5 ml. Each *Gc*LPMO9 enzyme was added to the fibers at a final concentration of 20 mg g^−1^ in the presence of 1 mM of ascorbic acid. Enzymatic incubation was performed at 40 °C under mild agitation for 48 h. Samples were then dispersed using a Polytron PT 2100 homogenizer (Kinematica AG, Luzern, Switzerland) for 3 min, and ultrasonicated by means of a QSonica Q700 sonicator (20 kHz, QSonica LLC., Newtown, USA) at 350-W ultrasound power for 3 min as previously described [20]. The reference sample was submitted to the same treatment but did not contain the enzyme. Wood cellulose fibers (reference and *Gc*LPMO9-treated) were deposited onto a glass slide and observed under a BX51 polarizing microscope (Olympus France S.A.S.) with a 4 × objective. Images were captured on a U-CMAD3 camera (Olympus, Tokyo, Japan).

## Abbreviations

α-MF: α-mating factor from *S. cerevisiae*
AA: auxiliary activity enzyme
CBM: carbohydrate-binding module
CMC: carboxymethylcellulose
PASC: phosphoric acid-swollen cellulose
*Gc*LPMO9: *Geotrichum candidum* AA9 LPMO
LPMO: lytic polysaccharide monooxygenase
XG: xyloglucan
